# Observation of the occupational scenario of community health workers

**DOI:** 10.47626/1679-4435-2021-735

**Published:** 2021-12-30

**Authors:** Beatriz Maria dos Santos Santiago Ribeiro, Marcia Eiko Karino

**Affiliations:** 1 Enfermagem, Universidade de São Paulo, Ribeirão Preto, SP, Brazil.; 2 Enfermagem, Universidade Estadual de Londrina, Londrina, PR, Brazil.

**Keywords:** community health worker, occupational health nursing, occupational health services, agente comunitário de saúde, enfermagem em saúde do trabalhador, serviços de saúde do trabalhador

## Abstract

Community health workers are inserted into family health developing activities of disease prevention and health promotion. These professionals need to recognize their work and the importance of using personal protective equipment. Observation revealed that some community health workers did not wear uniforms, and that their outfit included tank tops, skirts, and open shoes. Furthermore, there was no encouragement from the team nurse to the use of caps, sunglasses, sunscreen, long sleeve shirts, compression stockings, and closed shoes. These professionals were exposed to many occupational risks, such as ergonomic, physical, chemical, and biological risks, in addition to the risk of work accidents. This experience revealed three guiding axes. Community health workers had noticeable difficulties in identifying the appropriate use of personal protective equipment. Lack of training or qualification in occupational health was also observed; however, some community health workers had knowledge based on common sense or on personal experience, which has somehow influenced them in their precaution measures. The work preparation of these professionals is still precarious, being possible to perceive signs that their prevention of occupational risks is little remembered by managers. Therefore, further guidance should be provided to community health workers.

## Resumo

O agente comunitário de saúde está inserido na saúde da família desenvolvendo as atividades de prevenção de doenças e promoção de saúde. É necessário que esse profissional reconheça o seu trabalho e a importância do uso de equipamentos de proteção individual. Na observação, foi constatado que alguns agentes comunitários de saúde não utilizavam uniformes. Foi possível verificar que eram utilizadas roupas como regatas, saias e sapatos abertos. Percebeu-se que não havia incentivo pelo enfermeiro da equipe para utilização de bonés, óculos, protetores solares, camisas de manga longa, meias de compressão e sapatos fechados. São muitos os riscos ocupacionais aos quais esses profissionais estavam expostos, como riscos ergonômicos, físicos, químicos, biológicos e de acidentes de trabalho. Essa experiência elencou três eixos norteadores. Foi possível notar dificuldades dos agentes comunitários de saúde para identificar os usos adequados de equipamentos de proteção individual. Também foi evidenciada a falta de treinamento ou capacitação em saúde do trabalho; porém, alguns agentes comunitários tinham conhecimentos baseados no senso comum ou em experiências pessoais que, de alguma forma, os influenciaram na sua proteção. O preparo para o trabalho desses profissionais ainda é precário, percebendo, assim, indícios de que sua prevenção é pouco lembrada pelos gestores. Logo, são necessárias maiores orientações aos agentes comunitários de saúde.

## INTRODUCTION

The Family Health Strategy (FHS) aims for the initial reorganization of basic care, contributing for the organization of health care, qualification of access, welcoming, bonding, and continuity of care according to criteria of health need, vulnerability, and risk, among others.^1^ FHS teams need to face numerous challenges, such as the ongoing process of redefinition and qualification, focusing on ordination of care networks and effective health care management skills, by means of expanding actions and broadening the teams formats and the scope of actions to help increase resoluteness and coordination, with the support of other health care venues of the Health Care System.^2^

Community health workers (CHWs) are inserted into the FHS, developing activities of disease prevention and health promotion by means of individual and collective educational actions, both in households and in the community, under competent supervision. They are key players in the FHS, because they enable for population information and needs to be acknowledged by the team of health care professionals, who will intervene with the community.^3,4^ Therefore, it is crucial to gather information on the health of these workers.

Research on occupational health aims to study and intervene on the relationships between work and health, as well as to promote and protect workers’ health. Therefore, occupational health helps develop surveillance actions targeted at the risks existing in workplaces, working conditions, and occupational health problems, as well as organize and provide assistance to workers and understand diagnostic, treatment, and rehabilitation procedures in an integrated manner.^5^

Health care professionals should be advised and receive training to prevent accidents and occupational diseases. The use of personal protective equipment (PPE) is regulated by Regulatory Standard-6 (*Norma Regulamentadora*-6, NR-6), which describes that PPE consists of devices or products for individual use employed by workers and are designed to protect them from risks arising from threats to workers’ safety and health.^6^

Professionals need to perceive their work as a source of satisfaction and personal development, in addition to acknowledging the importance of using PPE to prevent harms to their own health. However, it is believed that describing this experience may contribute for the implementation of educational actions that may be useful in workers’ health care.^7^ Therefore, this study aimed to describe the experience of a graduate student in occupational health nursing with guidance to CHWs and with active observations about PPE.

## EXPERIENCE REPORT

This is a theoretical-descriptive, observational, and reflexive report, through a 2- month experience in the FHS, from December 2016 to January 2017, with the understanding that occupational health nursing is both an art and a science, which has a complexity that requires knowledge from the NR.^7^ The study was relevant due to the scarcity of observational reports and investigations targeted at this population.

The experience was conducted in a municipality in northern region of the state of Paraná, Brazil, which has three basic health units. All these units have FHS teams with 85% of coverage that assist a population of nearly 10 thousand inhabitants. The municipality had 24 CHWs; of which two were absent during this experience due to health conditions, and another three were absent because they were working in positions of “trust” with senior employees. Of those who were present at the time of the experience, 16 were women and three were men. Their working time in the FHS team ranged from 3 to 10 years.

In order to ascertain the labor activity of CHWs, this survey sought to gather information about how long CHWs worked in the FHS, as well as their age, schooling, and knowledge on the use of PPE, nursing supervision, and continuing education about their work.

CHWs worked in a schedule of 40 hours per week, 8 hours per day, starting at 8 a.m. up to 11.45 a.m., with a 75-min break for lunch, and resuming activities from 1 p.m. to 5 p.m.

The attributions of CHWs consisted of bureaucratic tasks, health surveillance, communication, health education, organization of demands, social support, and guidance to health prevention for the population of their micro-area, developing an extremely important social role, due to their bonds with the assisted families and to activities of health promotion, prevention, treatment, and rehabilitation. They also performed educational actions in schools, treatment and follow-up of patients with hypertension and diabetes, pregnant women, puerperal women, as well as home visits, among other activities relevant to their job and to the control of outbreaks of dengue mosquitoes.

Some CHWs did not wear a uniform. In the observation, it was possible to find that their outfit included clothes such as tank tops, skirts, and open shoes. It was noticed that there was no encouragement from the team nurse for CHWs to wear caps, sunglasses, sunscreen, long sleeve shirts, compression stockings, and closed shoes, as well as other PPE that could protect CHWs against occupational risks.

Their work instruments were a backpack weighting 5.3 kg, on average, in addition to pen, rubber, pencil, clipboard, and inkpot (to collect residents’ signature during visits as a proof for the team nurse, but some residents did not know how to read or write); CHWs also used measuring devices such as scale and measuring tape.

The home visit forms were entered into the electronic system of the Brazilian Unified Health System (e-SUS); each CHW had a day of the week in their schedule to enter their home visits into the e-SUS. The main mode of transportation of these professionals was walking, and some of them walked nearly 7 km a day. Noteworthy, CHWs did not perform home visits in the neighborhood where they lived, because the area of each CHW was assigned by “political promise”.

The FHS teams made sunscreen available to CHWs, which should be used every working day. These workers are exposed to many risks, such as ergonomic, physical, chemical, and biological risks, in addition to the risk of work accidents. After the first author provided training on occupational risks and use of PPE, and also on the importance of these issues to workers’ health, she chose three guiding axes from this experience, namely: practices and knowledge on occupational health; qualification on occupational health; and acknowledging the moments when CHWs neglected the use of PPE.

## PRACTICES AND KNOWLEDGE ON OCCUPATIONAL HEALTH

It was possible to note that CHWs reported to vocational training course directed to their occupation, although it was not a differing factor for the prevention and perception of occupational risks. However, they drove their reasoning to the fact that their previous courses did not address precautions about these risks and that occupational health has been left aside in the professional training of CHWs.

CHWs reported to be aware of methods to prevent sun exposure, mentioning that they were always careful, applied sunscreen, used an umbrella, wore a hat and closed shoes (since cases of dog bites were common in their municipality of work). It is noteworthy that some CHWs wore thin long-sleeved shirts in the heat, in order to protect themselves from the sun. However, some professionals did not apply sunscreen on cloudy days, because they believed it was unnecessary.

Therefore, it is essential for these professionals to be aware of the problem and to reflect on health at work, or the lack of it, as well as on the appropriate use of PPE. It is also bears highlighting the fact that participants reported to know only the basics on the need of PPE, and also reported not being aware of occupational diseases of their labor activity and had wrong knowledge, i.e., did not acquire appropriate knowledge.

## QUALIFICATION ON OCCUPATIONAL HEALTH

Despite the great variation in working time in the FHS, CHWs complained that they had not performed any course or training to use PPE and did not receive any guidance on occupational health from nurses of FHS or at the time of admission in the institution. However, they believed that training on occupational health was extremely important, because it is little provided in the municipality.

The author reminded of a CHW who reported that a cousin had skin cancer, and she had little information on how to prevent it. However, the nurse, the psychologist, and the social worker always conducted training sessions in companies on occupational risks, and this knowledge was then transmitted to the community. Nevertheless, it was not approached with CHWs from the FHS itself.

CHWs enjoyed the training session very much. This is a crucial point that should be reviewed by institutions, since the lack of training was a target of criticism from these professionals. Therefore, CHWs’ experiences showed that, although they had not received qualification on occupational health, they notice the demand for this qualification.

## ACKNOWLEDGMENT OF MOMENTS WHEN CHWS NEGLECTED THE USE OF PPE

During training, CHWs revealed lack of attention to health prevention, because some of them reported working wearing tank tops due to excessive sweat, whereas others said that their eyes were always irritated but they never remembered to use sunglasses. The use of flip-flops was common to “prevent foot blisters”; some CHWs did not use sunscreen because they complained it was oily, often acknowledging neglect and the risk for their health.

It was observed that CHWs were unprepared with regard to necessary precautions, because, even acknowledging their neglect, they often were unaware of some risks associated with occupational diseases. In this context, if CHWs had received guidance, they would know how to use of PPE, but, due to lack of instruction and to little knowledge on the risks of their work activities, CHWs state not knowing how to prevent work-related risks.

In this sense, it was possible to identify the need for providing information to CHWs about the use of PPE to prevent occupational diseases, and it is believed that these risks may be avoided by means of nursing guidelines to CHWs.

## DISCUSSION

Of note, there is the need to hear CHWs’ reports, because these professionals belong to the FHS team, which may prevent them from feeling undervalued, disqualified, and underpotentialized.^8^ It is worth mentioning that CHWs are able to provide differential care to the entire population, because they promote an improvement in people’s health by performing home visits, during which they use an accessible language and are able to establish relationship of dialogue and bond in their working routine.^9^ However, this only can be achieve if they are healthy and fit.

CHWs are mediator who coordinates health care actions between the health care team and the community.^10,11^ Therefore, their work routine involves technical actions related to the health guidance they provide, arising from the bond established with the communities in their area.^12^

A study indicated that CHWs had excellent knowledge on the comprehensive concept of health, overcoming the concept based mainly on disease and drug interventions.^13^ Thus, it is understood that their knowledge is related to life experiences or to common sense, since some of them reported not having any type of qualification on the subject. Nurses have great strategies to combine theory and practice, in addition to transmitting these strategies to CHWs by means of training sessions on the appropriate use of PPE.^14^

Based on these understandings, it is extremely needed to promote moments of exchange of health information within the team, in order to foster discussion and work instrumentalization,^12^ because recognizing the importance of care with one’s health and protection of workers’ health becomes necessary for an efficient health care. However, knowledge exchange helps solve health problems and is pointed in the research as one of the difficulties in professional practice.^12^

A study about CHWs concluded that they often worked with common sense, religion, and, less frequently, based on knowledge and resources from the families and from the community, because they did not have instruments and technology in the workplace. This scarcity makes them include knowledge for their work.^15^

The nurse who supervises the CHW is the main responsible for their training. However, it is not always provided, which is detrimental both to these workers and to users of the health care network, according to Ordinance 1886/GM, which approves the Standards and Guidelines of the Community Health Workers’ Program and of the Family Health Program.^16^ Hence, when the need for better protection of CHWs’ health is identified, supervising nurses are those who should offer themselves to provide appropriate training.^17^

As already mentioned, the Ordinance states part of the competence of instructor/supervising nurses to plan and coordinate CHWs’ training and permanent education,^16^ which are considered basic attributions that may be undertaken with the participation of other members of the health care professionals’ team from the local health service.^16^ Moreover, it was observed that CHWs did not lived in the neighborhood where they worked, which differed from what is suggested in guidelines, which aim to insert CHWs into their area of residence.^16^

In conclusion, CHWs experienced difficulties in identifying the appropriate use of PPE, because they should ideally use the required PPE for protection in their routine. There was also lack of training or qualification in occupational health; however, some CHWs had knowledge based on common sense or on personal experience, which somehow influences them in their precaution measures.

The work preparation of CHWs is still precarious, and it was possible to observed signs that prevention of occupational risks is little remembered by nurses in some FHS teams, being thus excluded from the planning of qualification and professional courses offered to CHWs. This report showed the need for providing further guidance to CHWs, as well as for promoting nurses’ qualification to provide training on basic occupational health actions, since nurses are considered CHWs’ instructors and responsible for their action and training.

Additional investigations on this theme are suggested, since some professionals are aware of the importance of using PPE and often did not use it appropriately because the matter did not receive due attention from their supervisors. The use of PPE should be encouraged, in order to reduce and prevent occupational diseases, since appropriate information and the health education of these workers are essential.
